# Analysis of outcomes of robot-assisted laparoscopic pyeloplasty in children from a tertiary pediatric center in South India

**DOI:** 10.3389/fped.2024.1376644

**Published:** 2024-06-26

**Authors:** Tamizhvanan Vidhya, Padankatti Rajiv, Venkat Sripathi

**Affiliations:** Department of Pediatric Urology, Apollo Childen's Hospital, Chennai, India

**Keywords:** RALP, pediatric, robotic surgery, prospective outcomes, PUJO

## Abstract

**Aim:**

This study aims to analyze the outcomes of robot-assisted laparoscopic pyeloplasty (RALP) in children with pelvi-ureteric junction obstruction (PUJO) over a 10-year period at a tertiary care center in South India.

**Methods:**

This study provides a detailed analysis of prospectively acquired data from 2013 to 2023 of all children who underwent RALP at our institution. Pre- and post-operative renal ultrasound and isotope renography were used to assess outcomes. Detailed information on patient demographics, procedural duration, post-operative pain relief, operation steps, and post-surgical follow-up protocols has been provided. The analysis included all patients who completed a 1-year follow-up.

**Result:**

Between 2013 and 2023, 201 children underwent RALP. Of these, 185 children completed at least 1 year of follow-up and were included in the analysis. The mean age of the cohort was 4.9 years (1 month to 17 years), with males comprising the majority (77.3%). Twenty-five children (13.5%) were younger than 1 year of age. Left-sided PUJO was found to be more common. The mean console time was 76.5 min (40–180 min), and the average hospital stay was 2.8 days (2–5). After surgery, the mean reduction in antero-posterior diameter of the renal pelvis was more than 50% of its pre-operative value and statistically significant (3.3 ± 0.3 to 1.9 ± 0.9 cm). At the end of 1 year, the overall reduction in renal size was also significant (9.7 ± 2.3 cm pre-operative to 8.9 ± 1.8 cm post-operative). The pre-operative Society of Fetal Urology (SFU) grade of hydronephrosis was compared to the post-operative SFU grade, and the improvement (resolution/downgrading) was found to be statistically significant. The median split renal function in this series was 39% pre-operative and 43% post-operative, and the overall functional improvement after RALP was significant. A successful outcome was observed in 181 children (97.8%). Four children experienced persistent severe hydronephrosis and underwent redo stenting and/or redo pyeloplasty (2.1% failure rate). Post-operative complications, according to the Clavien–Dindo classification, were classified as type 1 in three children and type 3b in two children. There were no conversions to open surgery in the series.

**Conclusion:**

RALP emerges as the minimally invasive procedure of choice for children with PUJO at our institution. It is safe, delivering consistently excellent results and minimal complications. Our outcomes are comparable to those of previously published series. We trust that our experience will serve as a roadmap for those centers (especially in South Asia) embarking on a pediatric robotic program.

## Introduction

Congenital pelvi-ureteric junction obstruction (PUJO) is the most commonly encountered obstructive uropathy in children. The Anderson–Hynes dismembered pyeloplasty is considered the “gold standard” surgical procedure for the correction of this problem. This procedure can be done by open, laparoscopic, or robotic approaches.

Open surgery has a success rate of 90%–100% (1). Although the laparoscopic correction of PUJO has a long history, the difficulty in intracorporeal suturing and knotting within a small space has prevented its widespread acceptance. On the other hand, robotic-assisted laparoscopic pyeloplasty (RALP) allows precise suturing within a timeline not very different from the open method. As the skills of robot-assisted laparoscopic suturing can be easily learned, RALP has gained enormous popularity.

Three-dimensional depth vision, increased degrees of freedom at the instrument tips, and motion scaling have made robot-assisted laparoscopy a very popular surgical option in India. Despite the large capital outlay in acquiring a robotic machine, many hospitals in India (both public and private) have commenced a robotic program.

Our center (in South India) pioneered robotic surgery in children in 2012, and in this paper, we review data acquired over a decade (2013–2023).

## Methods

At our institution, 201 RALP surgeries were performed on children between January 2013 and December 2023 (10 years). Of these, 185 children had completed a minimum of 1 year of follow-up (range 12–120 months) and were included in the detailed analysis. The database was prospectively maintained and retrospectively analyzed.

The objective of this review was to look at outcomes and complications so that it could serve as a roadmap for other centers in South Asia embarking on a pediatric robotic program. A detailed technical account is also provided.

Children with pelvi-uretic junction (PUJ) obstruction who underwent RALP and had completed at least 12 months of follow-up were included. Those within their first year post-RALP and those lost to follow-up were excluded from the analysis.

Our patient cohort included asymptomatic children whose antenatally detected hydronephrosis progressed to pelvicalyceal dilatation, along with a decrease in renal function and obstructive drainage patterns on ethylene dicysteine (EC) renogram. There were also cases presenting with urinary tract infection (UTI), hematuria, flank pain, and/or Dietl's crisis.

The pros and cons (including the cost) of open pyeloplasty and RALP were elaborately discussed, and the parents were allowed to make an informed decision. Laparoscopic pyeloplasty was not offered due to the lack of advanced laparoscopic suturing skills.

### Pre-operative investigations

Renal ultrasound and EC renal scintigraphy were mandatory investigations. The decision to surgically correct was primarily based on the Society of Fetal Urology (SFU) grade of hydronephrosis or an increase in the renal pelvic antero-posterior diameter (APD) on serial follow-ups, along with proven obstruction with or without any deterioration of renal function. In the presence of significant hydronephrosis, a palpable kidney, loin pain, urinary tract infection, or hematuria also served as valid indications for surgery.

### Operative protocols

#### Anesthesia, positioning, and port placement

**Figure 1:**
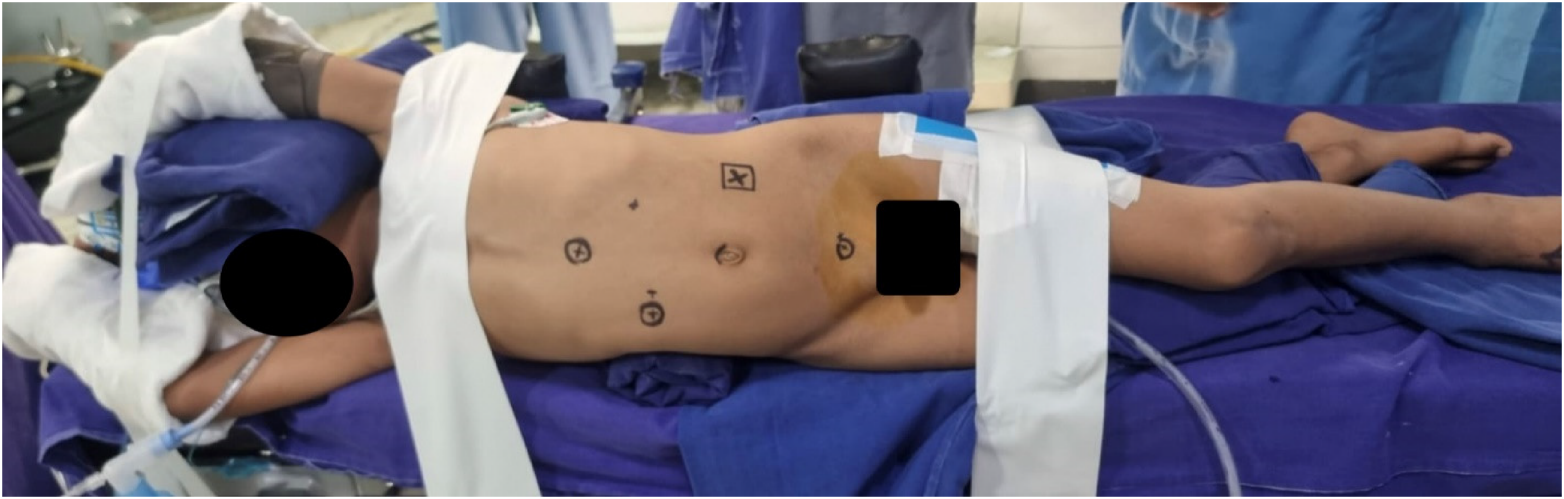
Child positioned for RALP.

**Figure 2:**
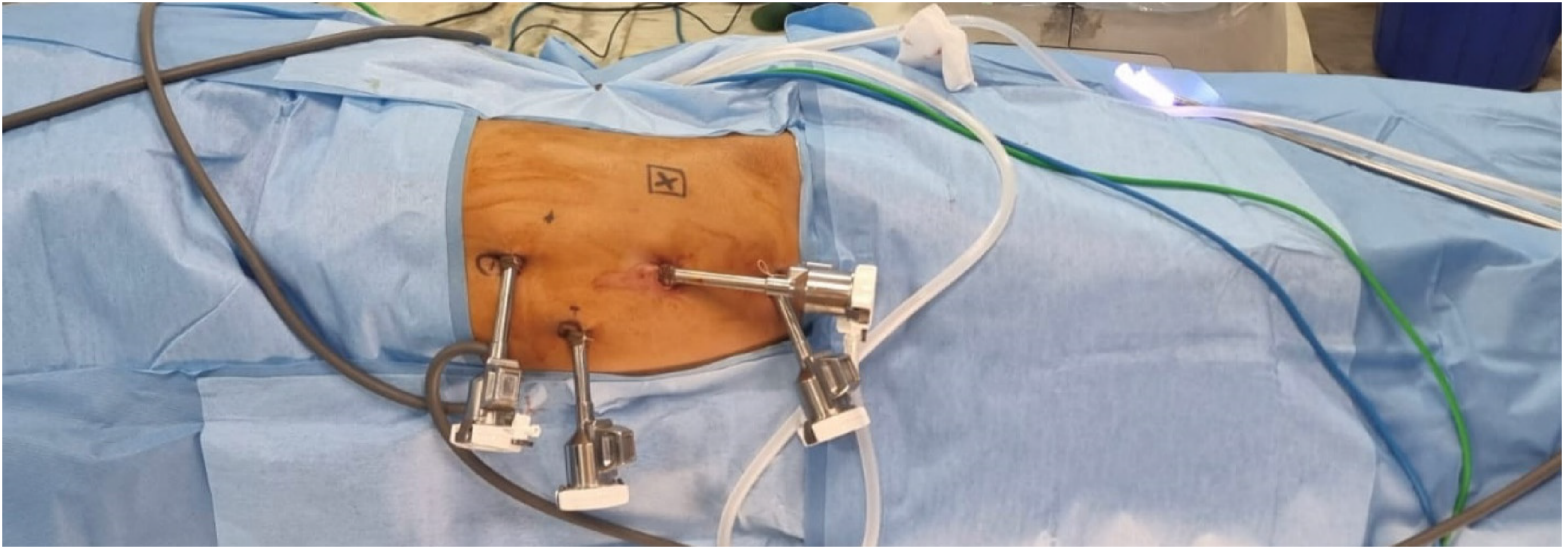
Port placement for RALP.

In children younger than 5 years of age, general anesthesia with a caudal epidural block (0.25% bupivacaine + 0.5 mg/kg morphine) was administered. In children older than 5 years, a single-shot lumbar epidural block was employed. The children were positioned in a lateral decubitus position at the edge of the table, with pressure points securely padded and limbs protected (Figure 1). The Da Vinci Xi robot was docked end to side. All ports were 8 mm in size and were placed in the midline, with the camera at the umbilicus and working ports in the epigastrium and hypogastrium. An 8-mm assistant port was placed either in the upper or lower quadrant, opposite to the side being operated upon and between the camera and one working port (Figure 2).

#### Steps of the procedure

The kidney was exposed by reflecting the colon medially (the retrocolic approach). In four cases, a trans-mesocolic approach was used because the PUJ could be easily exposed through an avascular mesenteric window. A 2-0 polyglactin (Vicryl) stay suture was introduced through the abdominal wall to hitch up the renal pelvis. This hitch-stitch was deliberately positioned high up on the dilated pelvis. This placement ensured that pelvic reduction could be accomplished without repositioning the hitch suture.

The PUJ was dismembered, keeping a pelvic cuff for atraumatic ureteral handling. The ureter was spatulated on the lateral aspect until vertical folds were visible and the lumen opened. The most dependent part of the pelvis was chosen, and a 5-0 Vicryl stitch was used to approximate the ureter and pelvis at the 6 o’clock position. This same suture was used to approximate the posterior pelvi-ureteric wall in a running manner. The tail end of this suture was left long. When commencing the anterior wall anastomosis, traction on this tail helped to define the lowermost end of the posterior wall anastomosis. The narrow segment of the ureter and excess pelvic cuff (used for traction) were excised, and the anterior anastomosis was completed with another running suture. All sutures were preferably 8–10 cm in length for ease of handling within a cramped space. A double J (DJ) stent was inserted at the commencement of the anterior wall closure. We did not find the need to stiffen the stent with a guide wire in any of our cases. Bladder placement was confirmed by the efflux of urine on suprapubic pressure.

**Figure 3:**
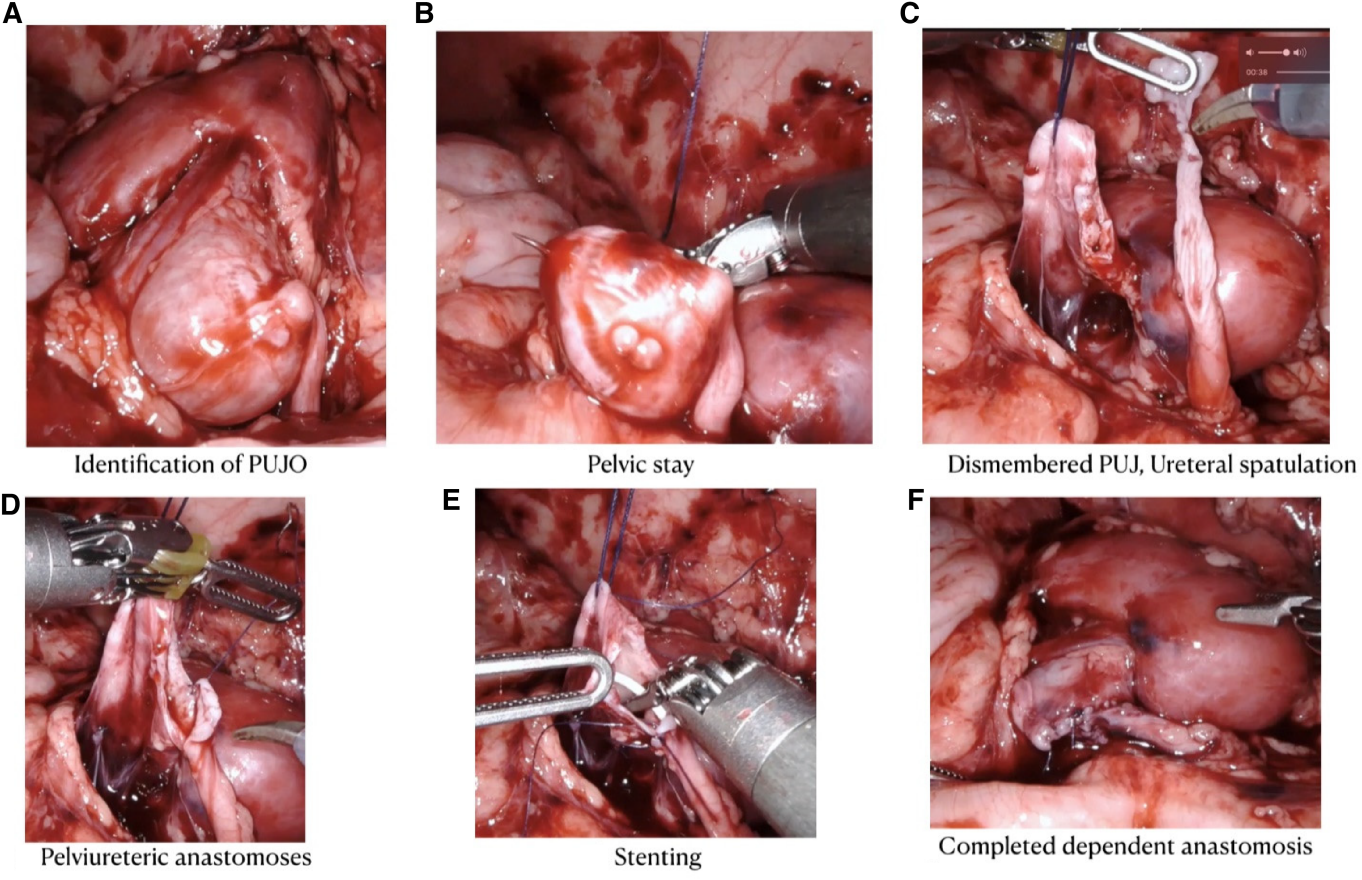
Intra-operative steps for RALP.

Trimming of a redundant pelvis was done from below upward (keeping the hitch-stitch in view). Pelvic closure was completed in a watertight manner after flushing out clots from the calyces and pelvis. In cases where the narrow segment of the ureter was more than 3 cm in length, a flap was swung down from the pelvis to reach the spatulated ureter. To fashion this flap, a radial cut was made at the largest diameter of the pelvis, and the lower portion was turned down and tubulated. This maneuver ensured that the ureter was not pulled upward. In the early part of our series, vigorous ureteral traction caused ischemia and a strictured PUJ, which needed resurgery (Figure 3).

#### Post-operative care

During the first 24 h, pain relief was provided by intravenous paracetamol, which was then switched to oral medication. Narcotics (intravenous tramadol 1 mg/kg) were used whenever the child had a pain scale score of more than 7 (visual analog scale). The urinary catheter was removed 72 h after surgery, and the child was discharged.

Most of our patients came from the northeastern part of India, with average travel times of 36 h by train. Therefore, parents preferred their children to be hospitalized until they were ambulant, pain-free, feeding well, and passing stools without any discomfort. This attitude toward hospitalization is unique to our population and demographics.

#### Post-surgical follow-up

One-month post-surgery, an in-person visit or a telemedicine review was scheduled to confirm that the stent was in position. Eight weeks post-surgery, cystoscopy and DJ stent removal were performed on a day-surgery basis. Again, the decision to retain stents for 8 weeks post-surgery was dictated by the long travel times.

Thereafter, ultrasound studies were conducted at intervals of 1, 3, 6, and 12 months post-surgery. At the 1-year review, a diuretic renogram scan was scheduled to assess drainage and function. If drainage was satisfactory, a review with ultrasound was performed a year later, and the child was discharged from further follow-up.

### Statistical analysis

Descriptive statistics are presented as frequency (%) for the categorical factors and mean ± SD for continuous factors. Skewed data are presented as the median [interquartile range (IQR)]. The Shapiro–Wilk test was used to determine the normality of the data. A paired sample *t*-test/Wilcoxon signed-rank test was used to determine the significant difference between pre-operative and post-operative visits. The McNemar–Bowker test was used to determine the proportional changes in hydronephrosis between pre- and post-operative visits. A *p*-value below 0.05 was considered statistically significant. All statistical analyses were performed using SPSS software (version 28.0, IBM).

## Results

### Demographics

The mean age of the patients in our series was 4.9 years (ranging from 1 month to 17 years). Twenty-five children were younger than 1 year of age. In total, 143 were males (77.3%), while 42 were females (22.7%). Left-sided PUJO was more common, occurring in 134 cases (72%) compared to 51 cases of right-sided PUJO (28%) (Table 1).

**Table 1:** Demographic factors.

Parameters	*n* = 185, *n* (%)
Age (years)	
Mean ± SD	4.9 ± 4.2
Median (IQR)	4 (1.5–7)
Range	1 month–17 years
Gender	
Male	143 (77.3)
Female	42 (22.7)
Side	
Right	51 (27.6)
Left	134 (72.4)

### Operative data

The total operation room (OR) time (wheels-in to wheels-out) was 158 min (range 145–240 min). The average console time was 76.5 min (range 40–180 min), and the average hospital stay was 2.8 days (range 2–5 days). Pelvic size reduction was performed in 60 cases. Thirteen children had a lower pole crossing vessel that was obstructing the PUJ. Two children had an incomplete duplex, with the PUJO confined to the lower moiety. In one case, the PUJO was seen in one horn of a horseshoe kidney (Table 2).

**Table 2:** Operative data.

Parameters	*n* = 185, *n* (%)
Console time	
Mean ± SD	76.5 ± 25
Median (IQR)	70 (60–90)
Range	40–180
Pelvic trimming	
No	125 (67.6)
Yes	60 (32.4)
Crossing vessel	
No	172 (92.9)
Yes	13 (7)

### Outcomes

Resolution of hydronephrosis and improvement in the drainage were observed in 97.8% (181) of the children. Complete resolution was noted in 26 children, and a significant change in the SFU grade was observed in 155 children. Four children needed reintervention due to persistent severe dilatation. Forty-three children did not undergo EC renogram, either because of complete resolution or because their parents were satisfied with the reduction in the SFU grade and did not favor another diuretic scintigraphy study. Of the remaining 142 children, a 5% increase in function was found in 88, while 12 showed a functional improvement of more than 10%. In 27 children, the pre-operative function remained unchanged. There was a 5% decrease in pre-operative function in 11 children (including four with supranormal pre-operative values), and a decrease of more than 10% was seen in 4 children.

The four children with >10% decrease in renal function and persistent severe dilatation were carefully followed up. Two underwent redo pyeloplasty, while two received long-term stenting to check whether drainage could be improved and function regained. Of the two children who underwent redo pyeloplasty, one subsequently underwent a ureterocalycostomy as a salvage procedure, resulting in a good outcome (>10% increase in function). Of the two children who underwent long-term stenting, one is yet to be evaluated and the other was lost to follow-up. These four children represent a failure rate of 2.1% in our series.

### Complications

Intra-operative complications included a partial renal vein injury during pelvic trimming in one child and a dropped needle, which was retrieved under C-arm guidance. Post-operative complications were classified according to the Clavien–Dindo scale. UTI occurred in three children post-surgery and was managed with oral antibiotics (type 1). There were two type 3b complications, which needed surgical correction. One child experienced omental herniation at the umbilical port site on the fifth post-operative day (POD). Another child presented with a grossly enlarged kidney, accompanied by pain and poor oral intake on the 10th POD, attributed to a blocked DJ stent. An emergency percutaneous nephrostomy was performed, followed by redo stenting. At 1-year follow-up post-surgery, this child showed complete resolution of hydronephrosis (Table 3).

**Table 3:** Complications (post-operative and intra-operative).

Post-operative complications: Clavien–Dindo classification	*N*	Type and management
Type 1	3	UTI treated with oral antibiotics
Type 3b	2	1. Omental hernia—repaired on POD5 2. Blocked DJ stent—PCN followed by redo stenting
Intra-operative complications	2	1. Partial renal vein tear—repaired 2. Lost needle in the abdomen cavity— retrieved using C-arm guidance

### Protocol 1 month after stent removal

The ultrasound findings at the 1-month review after removal of the stent were considered significant. If we found SFU grade 4 hydronephrosis with clubbed calyces, we deemed this a poor outcome. A retrograde pyelogram, with deployment of a long-term stent, was performed to enlarge the anastomosis and break the synechiae. If, after the removal of the redo stent, the hydronephrosis SFU grade and drainage did not improve, and there was a decrease in renal function, a redo pyeloplasty was necessary (open or redo RALP).

### SFU grading of outcomes post-surgery

Pre-operatively, the SFU hydronephrosis grade was 4 in 82 children (44.3%), 3 in 84 children (45%), and 2 in 19 children (10%). A post-operative ultrasound review after 1 year revealed the following: 26 children had complete resolution/SFU grade 1 (14.4%), 101 children had grade 2 (54.5%), 56 children had grade 3 (31.1%), and 2 children had grade 4 (1%). Post-RALP resolution and downgrading of hydronephrosis were found to be statistically significant (Table 4).

**Table 4:** Pre-operative and post-operative findings.

Parameters	Pre-operative	Post-operative	*p*-value
Hydronephrosis			
SFU 0–1	—	26 (14.4%)	**<0.001** ^a^
SFU 2	19 (10.3%)	101 (54.5%)	
SFU 3	84 (45.4%)	56 (31.1%)	
SFU 4	82 (44.3%)	2 (1%)	
Ultrasonogram APD (cm)			
*n*	185	185	**<0.001** ^b^
Mean ± SD	3.3 ± 1.3	1.9 ± 0.9	
Median (IQR)	3.3 (2.4–3.8)	1.9 (1.2–2.4)	
Range	1.2–10.8	0.5–5.2	
Ultrasonogram renal size (cm)			
*n*	185	185	**<0.001** ^b^
Mean ± SD	9.7 ± 2.3	8.9 ± 1.8	
Median (IQR)	9.3 (8–11)	8.5 (7.6–9.9)	
Range	5.6–19.1	5.8–15.2	
EC renogram			
*n*	185	142	**<0.001** ^b^
Mean ± SD	37 ± 12.5	39.5 ± 11.6	
Median (IQR)	39 (31.5–45)	43 (35–47)	
Range	1–85	0.1–83	

Boldface indicates statistical significance.

^a^ McNemar–Bowker test.

^b^ Paired sample *t*-test/Wilcoxon signed-rank test.

### AP diameter of renal pelvis 1 year post-surgery

The mean pre-operative APD was 3.3 ± 1.3 cm (range: 1.2–10.8 cm). One year post-surgery, the mean APD was 1.9 ± 0.9 cm (range: 0.5–5.2 cm). This difference was statistically significant (*p*-value <0.001) (Table 4).

### Renal size reduction 1 year post-surgery

The mean pre-operative size of the hydronephrotic kidneys was 9.7 ± 2.3 cm (range: 5.6–19.1 cm). At 1-year follow-up, the mean renal size was 8.9 ± 1.8 cm (range: 5.8–15.2 cm). The reduction in renal size after pyeloplasty, as documented by ultrasound, was found to be statistically significant (Table 4).

### Functional improvement

The median pre-operative split renal function was 39% (range: 1%–85%). One year after RALP, the median function was 43% (range: 1%–83%). Three children who showed supranormal pre-operative function (62%, 85%, and 83%, respectively) demonstrated function within the normal range at the 1-year evaluation. The overall functional improvement after RALP was statistically significant (*p*-value: 0.001) (Table 4).

## Discussion

RALP has emerged as the new gold standard in the management of PUJO in children. Several studies have proved its safety and efficacy, with success rates exceeding 90% (2–5). The outcomes evaluated include reduction of hydronephrosis, improved drainage and function, and symptom resolution. This paper analyzes prospectively acquired data of RALPs performed over a 10-year period (2013–2023) at a dedicated pediatric urology unit in South India. The mean age at surgery was 4.9 years (ranging from 30 days to 17 years), similar to that reported by Casale and Lambert (4.6 years) (6). Boysen and Gundeti et al. and Blanc et al. reported higher mean ages at surgery—6.8 and 9 years, respectively (2, 5). The study findings of a male preponderance (77%) and left-sided PUJO predominance (72%) along with other large series. Twenty-five of the 185 children were younger than 1 year of age.

Ultrasound and EC renogram were mandatory pre-operative investigations. All EC scans were conducted using the “F zero” protocol, in which the radio-isotope and furosemide are administered intravenously and simultaneously in a well-hydrated child. The kidneys were scanned for 20 min post-injection. The decision to operate was based on intra-renal transit time, the quantum of pelvic isotope retention in delayed images, and the assessment of the differential renal function. Since the study duration was only 20 min, T-half estimation was not relied upon. In an obstructed kidney with prolonged drainage, the T-half calculation involved a hypothetical extrapolation of the excretion curve, which we believed to be unreliable. Using the above protocol, we have never encountered a situation where the diagnosis of PUJO was proved erroneous during surgery.

In children presenting with lower pole crossing vessels (causing flank pain or Dietl’s crisis) or in situations of redo pyeloplasty, we preferred the F − 15 protocol, which overcame the limitations posed by the short scan time after isotope injection.

The approach to PUJO in 99% of the children in this series was transperitoneal and retrocolic. In a handful of cases, where the PUJ could be easily tackled through an avascular mesocolic window, this approach was used, especially if pelvic trimming was minimal. Dismembered pyeloplasty, using four ports of 8 mm size, was the norm. Using an 8-mm assistant port enabled the introduction and extraction of large sutures without straightening the needle, and completely eliminated the possibility of dropping the needle during transfer. In 48 children (25%), the length of ureteral narrowing exceeded 3 cm. To bridge the gap, a pelvic flap was swung down to complete the anastomosis. In children with lower pole crossing vessels, the PUJ was dismembered, the ureter was transposed anterior to the vessels, and anastomosis was completed. In poorly functioning renal units (<30% relative function), redundant pelves were trimmed to improve drainage. In every case, a DJ stent was employed and removed 8 weeks later by cystoscopy (a daycare procedure under general anesthesia). Although no perinephric drain was employed, we did not encounter any situation where the leakage of intraperitoneal urine was a problem following surgery. In our unit, RALP is mostly carried out by residents in training, with consultants providing guidance and taking over if needed. The mean console time in our series was 76.5 min, comparable to the timings reported by Casale and Lambert (82.3 min) and Blanc et al. (97 min) (6, 5). The total OR time from wheels-in to wheels-out was 158 min, comparable to the 173 min reported by Silay et al. in 185 patients. In Boysen and Gundeti’s study, the overall OR time varied between 160 and 363 min (2). The time spent on the console and the total OT time varies depending on the experience of the surgeon and the complexity of the procedure.

The average hospital stay in our series was 2.8 days. This is comparable to the 2.1 days reported by Silay et al. (7), 2.6 days by Murthy et al. (3), and 2.3 days by Lee et al. (8). Our unit attracts patients from other states (especially the northeastern part of India). Parents usually prefer to stay in the unit until the children are pain-free and ambulant for the long train journey back home. In keeping with this, we tend to remove the urinary catheter 72 h post-surgery (once hematuria has cleared and urine output has normalized). Parenteral analgesics are needed only for 24–48 h, and the use of narcotics has been limited to occasional older children with a low pain threshold. This approach is supported by studies indicating that RALP results in lower routine narcotic usage compared to open surgery (9, 10). Regardless of the surgical approach, the resolution of hydronephrosis is expected within 2 years post-surgery (11).

We compared the mean pre-operative and post-operative APDs of the renal pelvis. In the majority of cases, resolution was more than 50% and statistically significant. The criteria for defining the post-operative resolution of hydronephrosis vary from series to series. Carpenter et al. (12) reported no hydronephrosis as a success, while Rickard et al. (13) suggested that a pelvic APD of less than 15 mm is acceptable. Värelä et al. used an APD cutoff of less than 10 mm or a reduction of more than 50% of the pre-operative dimension as a criterion of success (11).

**Figure 4:**
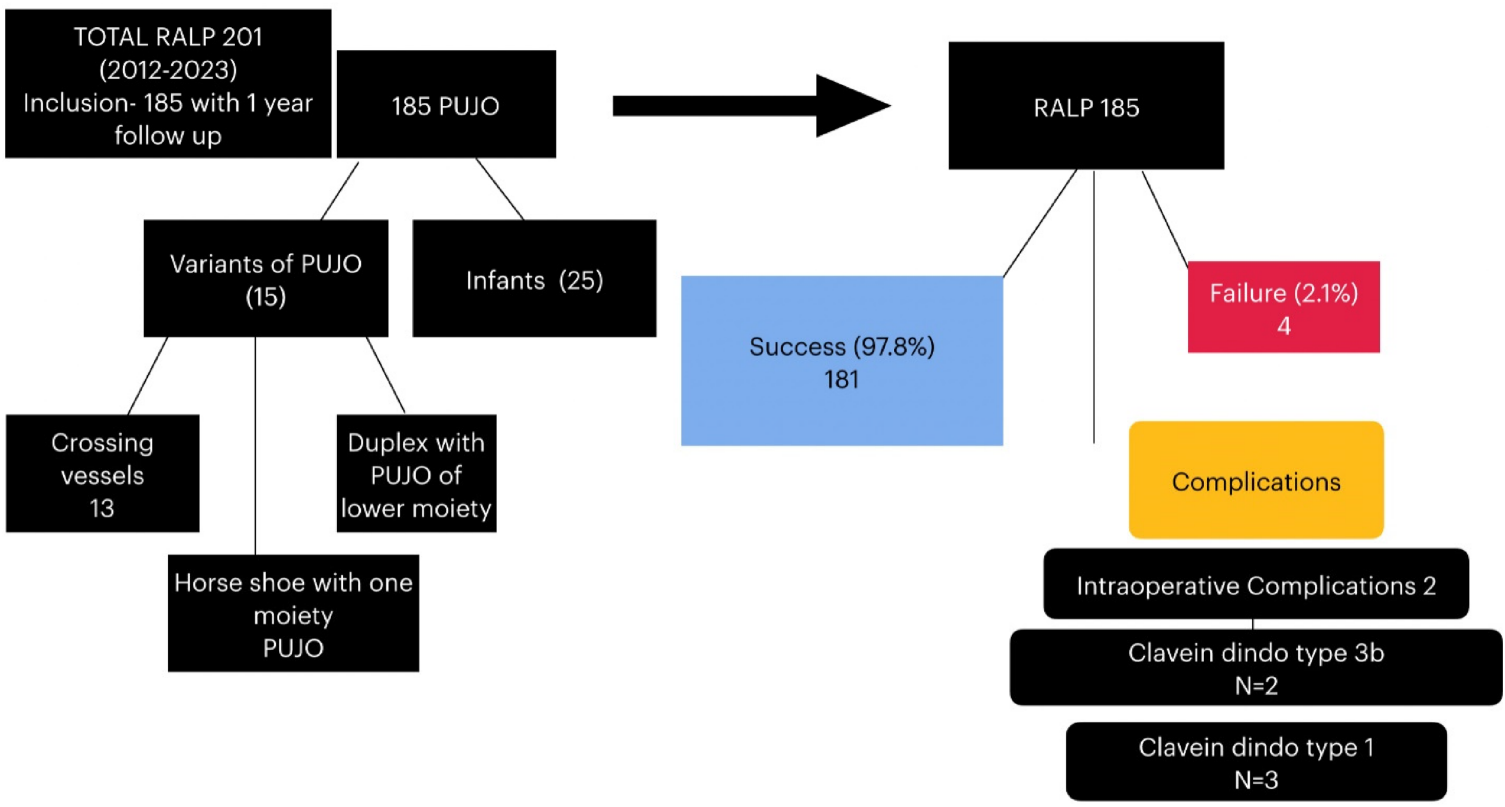
Overview of our outcome over a decade.

Renal size reduction was statistically significant 12 months after surgery (Table 3). Reduction in the overall renal size is an excellent indicator of good urine drainage and a successful pyeloplasty. The success rate of pediatric RALP ranges from 90% to 100% (2, 4, 5, 7). The largest single-institution study revealed a success rate of 96% and a reoperation rate of 3% (14–16). The success rate in our study is 97.8%, with a reoperation rate of 2.1%. The consistently excellent results of RALP are attributed to precision in the placement of fine sutures within a limited working space (Figure 4).

Dropping a needle when attempting extraction through a 5-mm assistant port made us change our assistant port size to 8 mm. This eliminated the need to straighten needles prior to removal, and we have not lost a single needle during transfer. A partial cut of the renal vein during pelvic trimming (which was repaired successfully) made us place the suspension suture very high up on the renal pelvis. This suture defined the limits of pelvis size reduction and served to protect flattened and stretched renal vessels from inadvertent injury.

**Figure 5:**
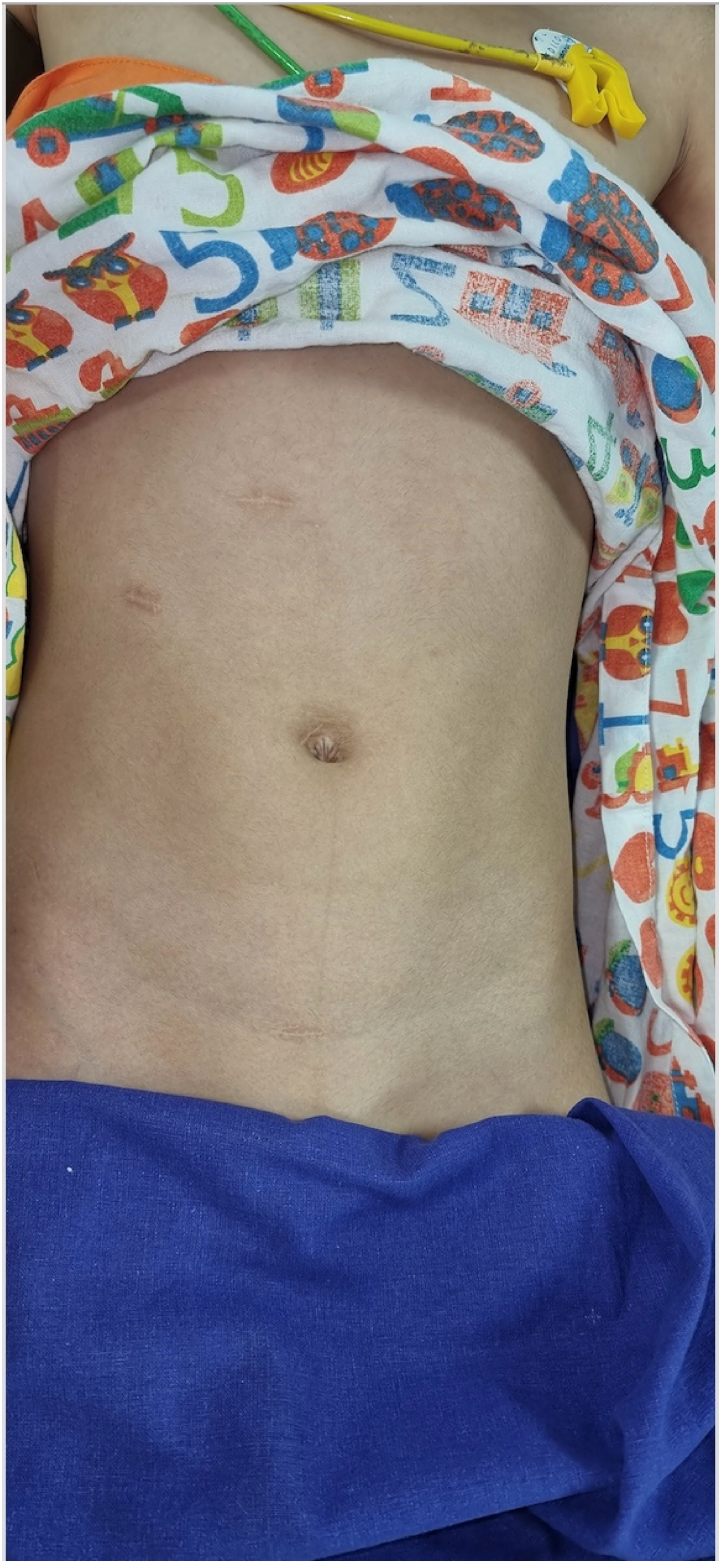
Cosmesis.

There were five complications in the early post-operative period. Three children developed UTIs requiring oral antibiotics (type 1 Clavien–Dindo). One child experienced an omental herniation needing resurgery, while another child suffered acute pain and renal swelling due to a blocked stent (type 3b Clavien–Dindo). Post-operative radionuclide studies demonstrated a median improvement of 4% in renal function (37% pre-operative to 41% post-operative), which was statistically significant (Table 3). Reduced pain, enhanced recovery, better cosmesis, and a good outcome are the prominent reasons for parents to opt for robotic surgery in children (Figure 5). This series is one of the few that has focused not only on improved drainage but also on differential functional improvement in the post-operative period.

## Conclusion

This is the first large series of pediatric RALP from South India, showing a success rate of 97.8%, which is on par with published series from the Western world. We believe that this report from our center will serve as a benchmark for other hospitals in India (and South Asia) that are embarking on a pediatric urology robotic program.

## Data Availability

The original contributions presented in the study are included in the article/Supplementary Material, further inquiries can be directed to the corresponding author.
